#  Mesoplasty with Pedanculated Seromuscular Flap in Type IIIb Jejuno-ileal Atresia 

**Published:** 2014-10-20

**Authors:** Ahmad Mohammadipoor, Nasrin Fatahi, Azin Malekmarzban

**Affiliations:** 1 Surgeon, Neonatal and Children Health Research Center, Golestan University of Medical Science, Gorgan ,Iran; 2 Pediatrician, Neonatal and Children Health Research Center, Golestan University of Medical Science, Gorgan ,Iran; 3 Researcher, Neonatal and Children Health Research Center, Golestan University of Medical Science, Gorgan ,Iran

**Dear Sir**

Jejuno-ileal atresia occurs as a result of an intra-uterine ischemic insult to the midgut. Type III b (apple peel) or (Christmas tree) deformity consists of proximal jejunal atresia, absence of the superior mesenteric artery, agenesis of the dorsal mesentery, a significant loss of intestinal length and a large mesenteric defect [1]. After the surgery, whether short bowel syndrome is occurred or not, it is important to begin enteral feeding as soon as possible. Short or absent true mesentery, coiling of distal small bowel around single vessel, and prevention of postoperative recoiling or volvulus are important challenges associated with type III b atresia besides short bowel syndrome. 

Since 4 years ago, we have had 5 neonates with jejunal atresia type III b who had been referred to our pediatric surgery center. Those infants included 4 boys and 1 girl who had gestational age ≥36 weeks and birth weight between 2100-2700 gr. They had no familial history and they were referred for surgery in first three days of birth. In order to decrease risk of volvulus, we have designed a method as following: 

In this technique, when apple-peel atresia was seen, a 4 cm segment of proximal atretic end of small bowel was isolated on vascular pedicle and opened with incision on the anti-mesenteric surface; thus the segment was converted to a pedunculated flap (Fig. 1). The mucosa of the flap was removed by cautery. This pedunculated seromuscular flap was sutured to the mesenteric defect after untwisting distal segment coils (the edges of the flap were sutured to the small intestinal mesenteric fat with Vicryl 5/0). End-to-end anastomosis was done. 

**Figure:**
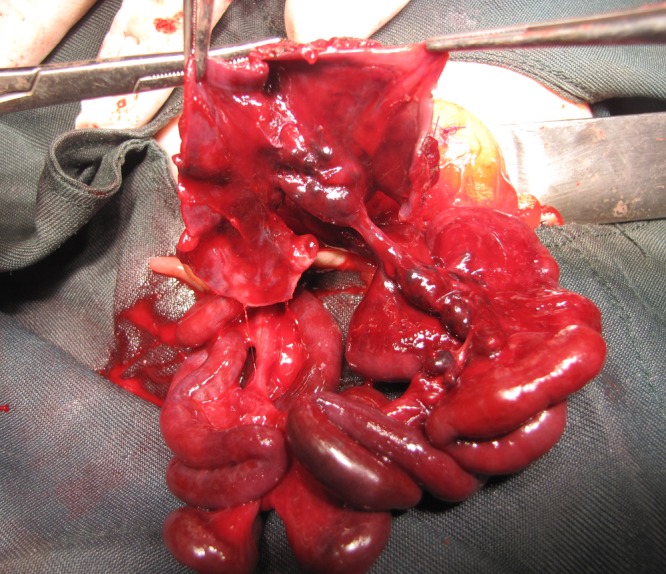
Figure 1: Preparation of seromuscular flap from isolated distal 4 cm of proximal atretic end of small bowel.

In all of our patients, postoperative course was uneventful. Interestingly in all patients we were able to start enteral feeding early. We followed patients for 2.5 years after surgery. One patient died at 13 months of age owing to the complications of short bowel syndrome and TPN. Other patients have normal weight for age percentile in their follow up and they had no problem up till now. There was no event of postoperative volvulus as previously reported by Lee et al.[2] The authors favor this technique for added benefits of early enteral feeding and prevention from possible postoperative volvulus.

## Footnotes

**Source of Support:** Nil

**Conflict of Interest:** None

